# Control of arm movements in Friedreich’s ataxia patients: role of sensory feedback

**DOI:** 10.1007/s00221-022-06343-5

**Published:** 2022-03-14

**Authors:** Lei Zhang, Andreas Straube, Thomas Eggert

**Affiliations:** 1 Institute for Neuroinformatics, Ruhr Universität Bochum, Universitätsstraße 150, 44801 Bochum, Germany; 2 Department of Neurology, Ludwig-Maximilians-Universität, Munich, Germany

**Keywords:** Reflex, Modeling, Movement disorder, Perturbation

## Abstract

Friedreich’s ataxia (FA) is a hereditary system degeneration, which progressively affects sensory functions such as proprioceptive feedback, which causes progressive ataxia in FA patients. While major clinical features of movement disorders in FA patients have been identified, the underlying impaired neural control is not sufficiently understood. To elucidate the underlying control mechanism, we investigated single-joint movements of the upper limb in FA patients. Small, tolerable force perturbations were induced during voluntary single-joint arm movements to examine the compensatory reaction of the FA patient’s motor system. Movement kinematics were measured, and muscle torques were quantified. We first found that as in healthy subjects, unperturbed single-joint movements in FA patients preserved similar temporal profiles of hand velocity and muscle torques, however, scaled in duration and amplitude. In addition, the small perturbations were compensated for efficiently in both groups, with the endpoint error < 0.5° (maximum displacement of 5–15°). We further quantified the differences in movement time, torque response, and displacement between patients and controls. To distinguish whether these differences were caused by a malfunction of top-down control or a malfunction of feedback control, the responses were fitted with a detailed model of the stretch reflex. The model simulations revealed that the feedback delay, but not the feedback gain was affected in FA patients. They also showed that the descending control signal was scaled in time and amplitude and co-contraction was smaller in FA patients. Thus, our study explains how the motor deficits of FA patients result from pathological alterations of both top-down and feedback control.

## Introduction

Friedreich’s ataxia (FA) is an autosomal recessive inherited disease that causes severe progressive movement disorders. The characteristic clinical features of this disease include gait and limb ataxia, dysarthria, and absent lower limb reflexes (Harding 1981; Cook and Giunti 2017). FA is caused by an abnormal amount of trinucleotide repeats in the frataxin (FXN) gene (Williams et al. 2021). Major sites of pathology in FA have been identified (Junck et al. 1994; Pandolfo 2008). Loss of large primary sensory neurons in the dorsal root ganglia leads to axonal sensory peripheral neuropathy, as well as atrophy of the posterior columns of the spinal cord, which causes loss of position and vibration sense. Atrophy of the spinocerebellar tracts, loss of proprioceptive input to the cerebellum, and the severe atrophy of the dentate nucleus are the cerebellar components of ataxia, leading to coordination disorders. Motor function is also affected by progressive degeneration of the corticospinal tracts affects. The resulting motor deficits of FA patients usually worsen over time and the patients become wheelchair-bound in their early twenties.

The human motor system has the essential feature of being able to maintain the stability of voluntary movements that are unexpectedly disturbed. In healthy subjects, the immediate reactions after such a disturbance include activation of short-latency spinal circuits as well as the intrinsic viscoelastic property of the muscles (Krylow and Rymer 1997; Pierrot-Deseilligny and Burke 2005). These low-level responses usually have a very short delay (< 45 ms in upper limbs). Supraspinal and voluntary reactions have a longer latency of 45–120 ms (Lee et al. 1982, Lewis et al. 2005; Pruszynski et al. 2008). The short-latency motor responses reflect extremely prompt feedback mechanisms, which highlight the role of proprioceptive sensory feedback in motor control. Although for large and destabilizing perturbations, the motor system must require high-level corrections to maintain the motor goal (Hasan 2005), peripheral feedback mechanisms may be sufficient against modest perturbations (Zhang et al. 2016). For example, if subjects are instructed not to voluntarily act against a transient and small perturbation, they can reach the same final arm position (Jaric et al. 1999; Rothwell et al. 1982; Schmidt and McGown 1980) due to the automatic peripheral feedback mechanisms.

In FA patients, these low-level feedback mechanisms, in particular, spinal stretch reflex are usually examined during posture by recording the surface electromyogram in leg muscles during sudden perturbations and the main finding is a delay of these stabilizing reflexes (Diener et al. 1984). While clinical signatures imply that FA patients may have impaired posture stabilization, the contribution of such reflexes to upper limb movement stabilization is not well understood and it has not yet been sufficiently investigated to what extent short-latency reflex mechanisms may contribute to the stabilization of upper limb movements in FA patients. Considering that one of the clinical features of FA is a general degeneration of peripheral sensory nerves involved in different parts of the body (i.e. sensory polyneuropathy), we expected a deficit or abnormal patterns in short-latency feedback in these patients. Feedbacks with longer latencies may also be affected in FA patients, however, testing this is beyond the scope of the current study.

Considered as the disease hallmark of FA, ataxia is usually associated with prolonged movement durations and lower velocities in both single and multi-joint movement measurements (Ramos et al. 1997; Topka et al. 1998). However, such analyses of upper limb movements are not widely available to clinicians (Maring and Croarkin 2007), and a more accessible and precisely quantifiable measurement is needed.

In our study, we established an experimental setup to enable the measurement of kinematics with computer-controlled force perturbations during single-joint arm movements in FA patients. Based on these measurements, we developed a data analysis approach to reveal the functional significance of neural control loops by comparison between these patients and healthy controls. We focus on the function of short-latency peripheral mechanisms in movement generation and in coping with unexpected mechanical perturbations, which challenge the motor system’s stability in FA patients. Small, tolerable force perturbations were induced during voluntary arm movements to examine the stability of the FA patient’s motor system. We quantified the differences in movement time, torque response, and displacement between patients and controls, which may be related to modified top-down movement control. To disentangle such differences of top-down control from impaired feedback-control, a detailed model of how the stretch reflex acts during such movement was implemented (Feldman and Levin 1995). Comparing model simulation results of both healthy subjects and FA patients provided insights into neural mechanisms for system stability, i.e., the compensation for small perturbations during single-joint arm movements.

## Methods

### Subjects

Six FA subjects (four males and two females, mean age: 50.7 ± 9.4 yrs, range 39–61 yrs, genetically confirmed FA, right-handed) participated in this study. Before the participation, they were all accessed with a standard neurologic examination (Campbell 2012), which was performed by clinicians at the University Hospital Munich, Germany. Patients were also screened for a pharmacological study with a systematic assessment (Reetz et al. 2019). Four of them had light dysarthria/brady-dysdiadochokinesis of the tongue and the other two had macro-square wave jerk during fixation, while other potential malfunctions of the cranial nerve were not observed. Their arm muscles did not show hypotonia or manifest paresis or atrophies, examined by passive flexion and extension of the limbs. Muscle tendon reflexes in lower limbs were absent in all patients. For most of them (except one patient), hand fine motor skills and finger follow-up (tested by hand rapid alternating movements, finger-to-nose maneuvers, follow-up of the fingertip of the examiner who changes finger position in a jerky manner, etc.) were impaired. Sensibilities such as aesthesia, algesia, and thermesthesia were normal, while three subjects had bimalleolar pallesthesia (score under 6/8, rated with Rydel-Seiffer tuning fork). Although most of them could stand independently, they all had deficits in walking. The average SARA score was 9.0 ± 2.9 (of 40). The patients did not have cognitive deficits (MOCA test range 26–29 out of 30).

Six healthy subjects (four males and two females, mean age: 34 ± 10 yrs, range 29–54 yrs) served as controls. They were all right-handed and did not suffer from any movement disorders or neurological diseases (self-report). Both healthy and FA subjects were naïve with respect to the purpose of the study. The experimental procedure was in accordance with the Declaration of Helsinki and approved by the local Ethics Committee. Written consent was obtained prior to participation in the experiment.

### Apparatus

**Fig. 1:**
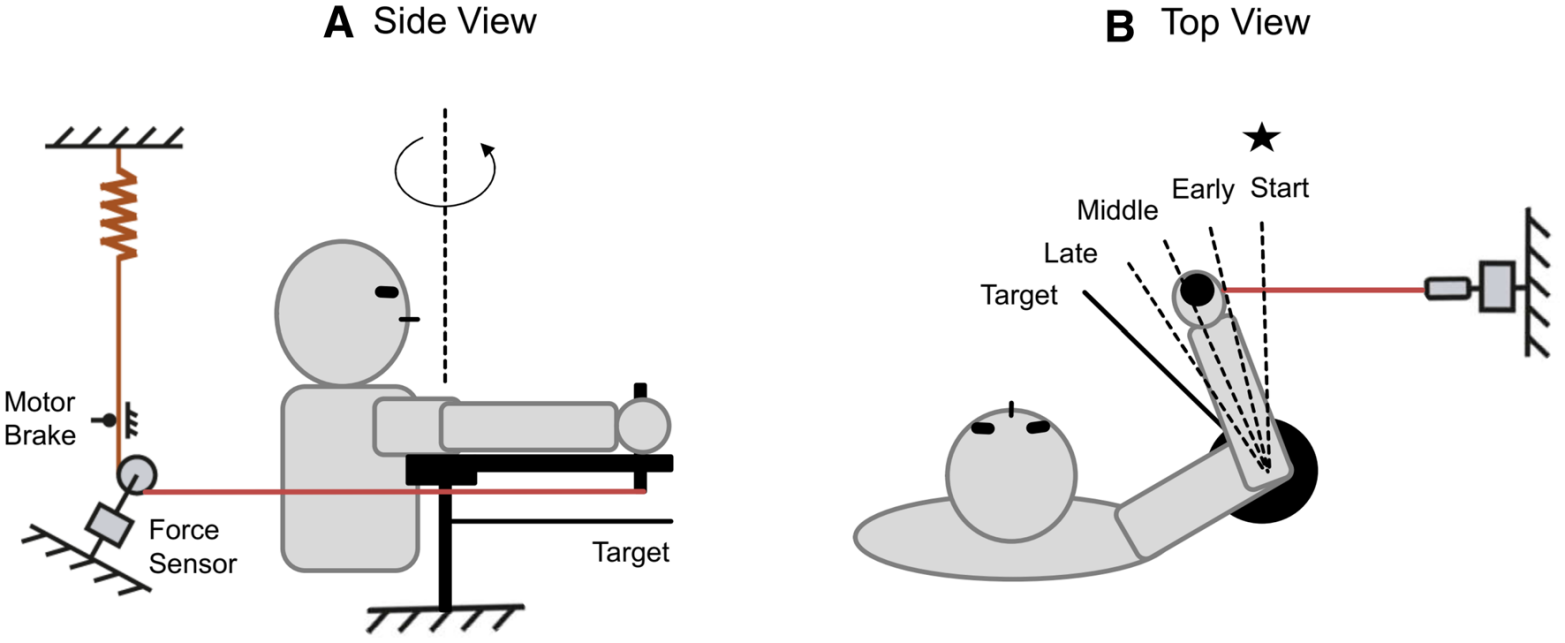
Experimental Setup. **A** Side view. Elbow joint movement was recorded by an ultrasound marker placed on the top of the grasping handle. The mechanism of the perturbation apparatus is described in the text. The pulling direction of the rope was to the right of the subject (see the top view in (**B**)) and it was modified here in the side view for clarity. **B** Top view. The movement started at an elbow joint of 60 deg extension (full extension: 0 deg) where the subject was instructed to align the forearm with an external marker (the filled star). The subject made a fast elbow flexion to the target (solid bar), which was at the elbow position of 105 deg. An impulse-like perturbation could be triggered at an early (about 25%), middle (about 50%), or late (about 75%) stage of the movement

The apparatus shown in Fig. 1 was used to investigate the arm response to external perturbations. The subject sat on a chair and the elbow joint was supported by a plate. The plate was about 1 m above the ground and mounted on the vertical rotary shaft of a ball bearing. The height of the chair was adjusted so that the subject’s forearm could move in a horizontal plane passing through the shoulder. The elbow joint was aligned with the pivot of the rotatable shaft and the forearm was fixed to the plate by a velcro tape. A grasping handle was mounted on a metal bar for forearm support and was adjusted to the forearm length. In the start position, defined by an external marker (see Fig. 1), the elbow joint angle was flexed by about 60 degrees (full extension 0 degrees). A thin horizontal bar below the arm served as the target position at an elbow angle of 105 deg. Visual information is known to influence the online updating of motor commands during movement execution (Sarlegna and Mutha 2015). Thus, to prevent visually guided corrections, visual feedback during the movement was excluded by asking the subjects to close their eyes before the movement started and open them again when the movement ended.

Limb movement was recorded by a 3D ultrasound-based motion analysis device (Zebris Medical GmbH, Isny, Germany). One marker was attached to the grasping handle and was sampled at 200 Hz. The perturbation was applied by a high stiffness rope attached to the grasping handle. The pulling direction of the rope was in the same horizontal plane as the forearm. The other end of the rope was connected to a low-stiffness spring, which was fixed to the wall (Fig. 1B). The middle part of the rope went through a brake, where the rope could be suddenly jammed by a metal pin that could be pulled onto the rope using an electric magnet. The magnet was controlled by a digital signal switching the magnet on or off. When the magnet was switched off, the rope was released and could pass freely through the brake. The force in the rope was measured by a strain gauge that allowed accurate dynamic force measurement (Rieger, Rheinmünster, Germany).

A computer with a real-time experimentation and recording system REX (Hays et al., 1982) received the analog data online from the Zebris device and the force sensor and mapped all these data into a common sampling frame (1 kHz). The resolution of the A/D measurement board was 16 bits with an amplitude range of ± 10 Volts.

### Procedure

Subjects were instructed to move the right arm from the start position to the target position in a single fast movement (45 deg elbow flexion). If the arm was perturbed during the movement, the subject was instructed to ignore the perturbation and not to correct it in case the target was missed. Before each trial, the subject pressed a button to initiate the trial. A beep, generated at a random time interval between 1.3 and 2.3 s after the button press served as the go signal for movement initiation. Immediately after the beep, the subject closed their eyes and initiated the movement. The subject’s arm stayed at the movement end position until the eyes were opened. Then, the subject returned to the start position and prepared for the next trial.

There were two trial types: unperturbed and perturbed trials. For perturbed trials, the magnetic brake was triggered when the position signal of the marker exceeded 25% (early), 50% (middle), or 75% (late) of the distance between the start position and the target. The duration of the braking was 100 ms and the corresponding torque perturbation along the movement direction was brief and small. Perturbation torque in our setup was triggered by a brake mechanism, which did not completely block the rope in the brake but induced transient friction. Consequently, force- and torque-amplitude increased with increasing movement speed at trigger time. Across all participants, the average peak torque amplitude of all trials was about 2.5–3 Nm for the early and middle conditions and about 2.2 Nm for the late condition. The shape of the perturbation time course was nearly symmetric around its peak. Perturbation durations were about 150 ms for early and middle trigger conditions and about 80 ms for the late condition. During unperturbed trials, the rope could move freely, and the preloads induced by the low-stiffness spring stayed below 0.8 Nm.

Before the main experiment, each subject performed 30 practice trials (mixed trial types and trigger conditions, without vision during the movement) so that they could familiarize themselves with the experiment. The main experiment consisted of 150 trials in total, including 36 perturbed trials. The perturbed trials consisted of 12 trials of each trigger condition (early, middle, or late) that occurred in random order. The other 114 trials were unperturbed. All perturbed trials were randomly interspersed among the unperturbed trials to avoid prediction of the perturbation. At least two consecutive unperturbed trials followed a perturbed trial, and later for the kinematic analysis, any unperturbed trial that appeared right after a perturbed trial was discarded to avoid any possible one-trial adaptation (Weeks et al. 1996). After this exclusion, 78 unperturbed trials were included in the analysis. After every 50 consecutive trials, the subject took a break for 5 min to prevent muscle fatigue.

### Kinematic analysis

The elbow joint angle was reconstructed from the marker position. The zero position was defined as the configuration at the start position. The time course of the elbow joint angle was first interpolated in 1 kHz and then filtered by a symmetric (zero-phase) Gaussian low pass with a cut-off frequency of 30 Hz. A three-point differentiator was subsequently applied to obtain elbow angular velocities.

Movement start was defined as the time when velocity first reached 10% of its peak and movement end was defined as the time when velocity decreased to 10% of its peak. For every subject, an outlier analysis was performed separately for both trial types, based on the acceleration interval, i.e., the time between movement start and the peak velocity. Trials with an acceleration interval that differed by more than twice the interquartile range (25–75%) from the mean of the respective trigger condition were excluded as outliers. On average about 1 (healthy, range 0–2)/3(FA, range 1–6) perturbed trial out of 36 and 3(healthy, range 1–6)/5(FA, range 2–7) unperturbed trials out of 78 were excluded.

To quantify the consequence of the perturbation and how efficiently the motor system compensated for this effect, the joint displacement, defined as the time course of the position difference between perturbed and unperturbed trials, was analyzed. Displacement speed was defined as the velocity difference between two trial types (i.e. the derivative of displacement). The onsets of the displacement or the displacement speed were defined as the time when they first differed significantly from zero. To quantify the effect of the perturbation on the end position of each movement, we evaluated the displacement at 100 ms after movement offset.

### Limb dynamics

The dynamic model of the limb consists of two segments: the hand was modeled as a ball and the forearm was modeled as a frustum (which capture arm geometric shapes and have been used to predict their inertial properties, e.g., Agarana et al. 2017). The inertia of the two limb segments for each subject was determined using anthropometric data (Winter 2009) based on the measurements of the subject’s weight and height. The limb dynamics were computed by the following equation:

1 $$\mathrm{I}\ddot{\uptheta }={\uptau }_{\mathrm{m}}+{\uptau }_{\mathrm{ext}}.$$

The total inertia I, including the inertia of the hand, the forearm, and the apparatus, was 0.103 ± 0.022 kgm^2^ (healthy and FA). The elbow joint angle θ is 0 deg at the start position and increases when the elbow flexes. τ_m_ is the joint torque caused by muscle torques and τ_ext_ is the joint torque corresponding to the force that is externally applied by the rope.

Torque response was defined as the muscle torque (i.e. τ_m_ in Eq. 1) difference between perturbed and unperturbed trials. Torque response onset was defined as the time when the positive torque response first differed significantly from 0.

### Simulations

The simulation was based on a threshold position control model (Pilon and Feldman 2016; Zhang et al. 2016). According to this model, the brain sets a control command as the threshold muscle length, at which a muscle becomes active. Muscle activation depends on the difference between the threshold length and the actual muscle length. A characteristic feature of this model is that the setting of the threshold is a pure feedforward command, whereas muscle activation and force, as well as kinematic change result from the interaction between all physical elements such as reflexes, proprioceptive feedback, and the biomechanics of muscles and environment. The model considers the case of one symmetric agonist–antagonist pair of muscles with a single joint (flexion/extension motion). Two types of control commands were defined: one specifies the position (R) at which both groups may be silent (a common threshold position) and the other specifies the spatial range (2C, i.e. the difference between threshold positions of symmetric antagonistic muscles, while C is the co-activation command to each muscle, see below) within which these muscles may be co-active. The descending commands for the flexor and the extensor (called threshold muscle length) are defined by the combination of R and C. A muscle is activated if the difference between the current muscle length and the velocity-dependent threshold muscle length is positive. Several peripheral neural constraints and muscle properties are considered (more details on model equations and parameters in Appendix 1).

To evaluate the descending control and peripheral constraints in both healthy and FA subjects, we performed a model parameter optimization procedure. Five model parameters/variables were fitted for each patient and each control subject. Three parameters were related to the descending control: (1) *Start time*: onset of control commands with respect to movement onset; (2) *RC time*: duration of control commands; (3) *C amplitude*: level of co-contraction. Two parameters characterized the sensorimotor feedback: (4) *u*: reflex gain. (5) *Delay*: reflex delay. The optimal values of five variables were found using constrained non-linear optimization (fmincon function), by minimizing the cost function of the sum of square error between simulated and recorded arm trajectories in all conditions (no/early/middle/late perturbation). For this optimization procedure, the parameter constraints, as well as the appropriate starting values of the fitted parameters, are described in Appendix 2. This section also shows a sensitivity analysis that assesses the quality of the model fit. Implementation and simulation of the model, as well as the optimization and all other data analysis was done in Matlab (R2020b)/Simulink.

### Statistical analysis

Descriptive statistics were reported as the mean and standard deviation in the text and the table, and as the mean and the 95% confidence interval of the mean in the figures. For experimental data, the onset times of displacement, displacement speed, and torque responses, as well as maximal values of displacement and torque responses were compared between healthy subjects and patients using unpaired *t* tests. To evaluate the quality of fit, the time courses of joint positions were compared between experimental and simulated data using the squared coefficient of the correlation, and the mean square residuals were provided. Comparison of movement and model parameters between the healthy subjects and patients was done with unpaired *t* tests.

## Results

### Unperturbed movements

**Fig. 2:**
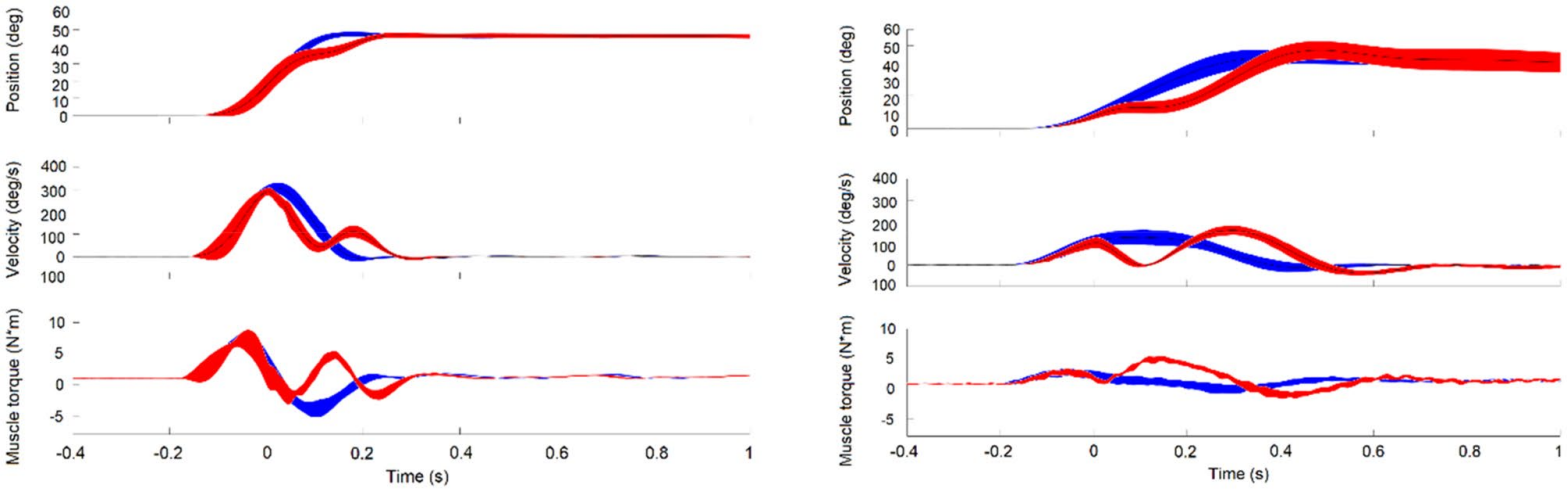
Experimental results of one healthy subject (left) and one FA patient (right) with 95% confidence interval (shaded area) for the trigger condition early (onset of perturbation shortly before peak velocity). Position, velocity, and muscle torque are shown for unperturbed (blue) and perturbed trials (red). Time 0 is perturbation onset

The kinematics of unperturbed movements (trigger condition: early) for one sample healthy subject and one FA patient are shown in blue in Fig. 2, and the characteristic movement parameters averaged across subjects are provided for both groups in Table 1. Overall, the FA group had a longer movement duration and a smaller peak velocity and torque. Although these kinematic and dynamic movement parameters differed between the two groups, the relative shapes of the kinematic and dynamic profiles, e.g., the timing of peak velocity and torque values with respect to movement duration, were similar (Table 1). For both groups of healthy and FA subjects, the movements showed a bell shape velocity and a bi-phasic torque profile (blue curves in Fig. 2).

**Table 1:** Experimentally observed movement characteristics of unperturbed movements (mean ± SD) in healthy and FA subjects (*n* = 6)

Measure	Healthy	FA patient	*t *test
Movement duration (ms)	296.0 ± 63.3	841.7 ± 354.7	*t*(5) = − 3.71, *p = *0.004
Time of peak velocity (ms)	136.3 ± 36.9	343.7 ± 118.3	*t*(5) = − 4.10, *p = *0.002
Time of peak velocity (% of movement)	45.8 ± 4.1	42.1 ± 5.36	*t*(5) = 1.36, *p = *0.204
Time of first peak torque (ms)	54.9 ± 17.3	142.6 ± 106.6	*t*(5) = − 1.93, *p = *0.083
Time of first peak torque (% of movement)	19.9 ± 2.7	16.1 ± 4.9	*t*(5) = 1.68, *p = *0.125
Time of second peak torque (ms)	217.6 ± 54.5	430.4 ± 117.4	*t*(5) = − 4.05, *p = *0.002
Time of second peak torque (% of movement)	73.3 ± 5.7	59.6 ± 27.9	*t*(5) = 1.179, *p = *0.266
Peak velocity (°/s)	275.0 ± 62.0	87.7 ± 28.6	*t*(5) = 6.72,* p* < 0.001
First torque peak (Nm)	5.00 ± 1.58	2.47 ± 0.44	*t*(5) = 4.26, *p = *0.002
Second torque peak (Nm)	– 2.74 ± – 1.38	0.75 ± 0.47	t(5) = − 6.13, *p* < 0.001

The times of peak velocity, 1st and 2nd peak muscle torques are expressed with respect to movement start

### Perturbed movements and perturbation responses

The kinematics of perturbed movements for the early trigger condition of one healthy subject and one FA patient are shown in red in Fig. 2. For both subjects, the joint angle was diverted by the perturbation and then converged back to the unperturbed one (in blue). The perturbed movement almost stopped and reaccelerated to reach the target. In both subjects, after perturbation onset (Time 0), the muscle torque showed a second positive peak (in red), and this deviation from the torque in the unperturbed condition (in blue) was characterized as torque response (see Methods). Similarly, the differences in position (Fig. 2 first row) and velocity (Fig. 2 second row) were characterized as displacement and displacement speed (see Methods).

**Fig. 3:**
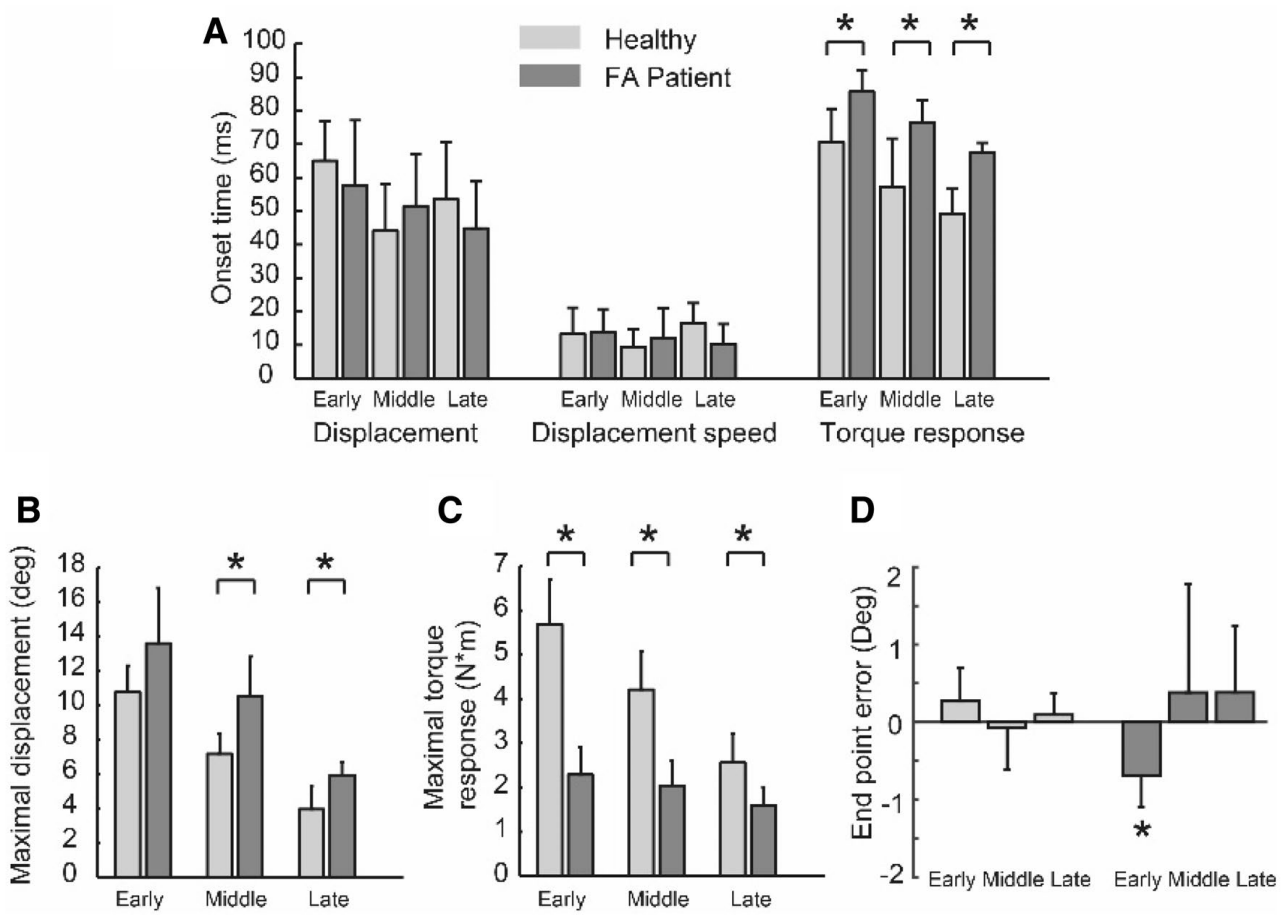
Comparison of perturbation effect in healthy and FA subjects (*n* = 6). **A** Onset times of displacement, displacement speed, and torque response in two groups. **B** Maximal displacement **C** Maximal torque response **D** End point error. Asterisks indicate significant differences (*t* test: *p* < .05)

The onset times of displacement, displacement speed, and torque response with respect to perturbation onset for both groups are summarized for all trigger conditions in Fig. 3A. The onset times (relative to perturbation onset) of the displacement and displacement speed across conditions did not differ between the two groups (for displacement: all *p *values > 0.46, for displacement speed: all *p* values > 0.19, Fig. 3A). The onset times of torque response in FA patients were larger than those in healthy subjects (*p* < 0.039 for all trigger conditions, Fig. 3A). Maximal displacement in both middle and late conditions was larger in FA patients than in healthy subjects (middle: *p = *0.031; late: *p = *0.030), but it did not differ between the two groups (*p = *0.151, Fig. 3B) in the early condition. In all conditions, healthy subjects had a larger maximal torque response than FA patients (all *p* values < 0.034, Fig. 3C). For both healthy and FA subjects, the averaged endpoint error was smaller than 1 deg (Fig. 3D). In the early condition FA patients arrived short of the target (*p = *0.022), while in other conditions, the endpoint error did not differ from zero in both groups (all *p* values > 0.267, Fig. 3D).

### Model parameter optimization

**Fig. 4:**
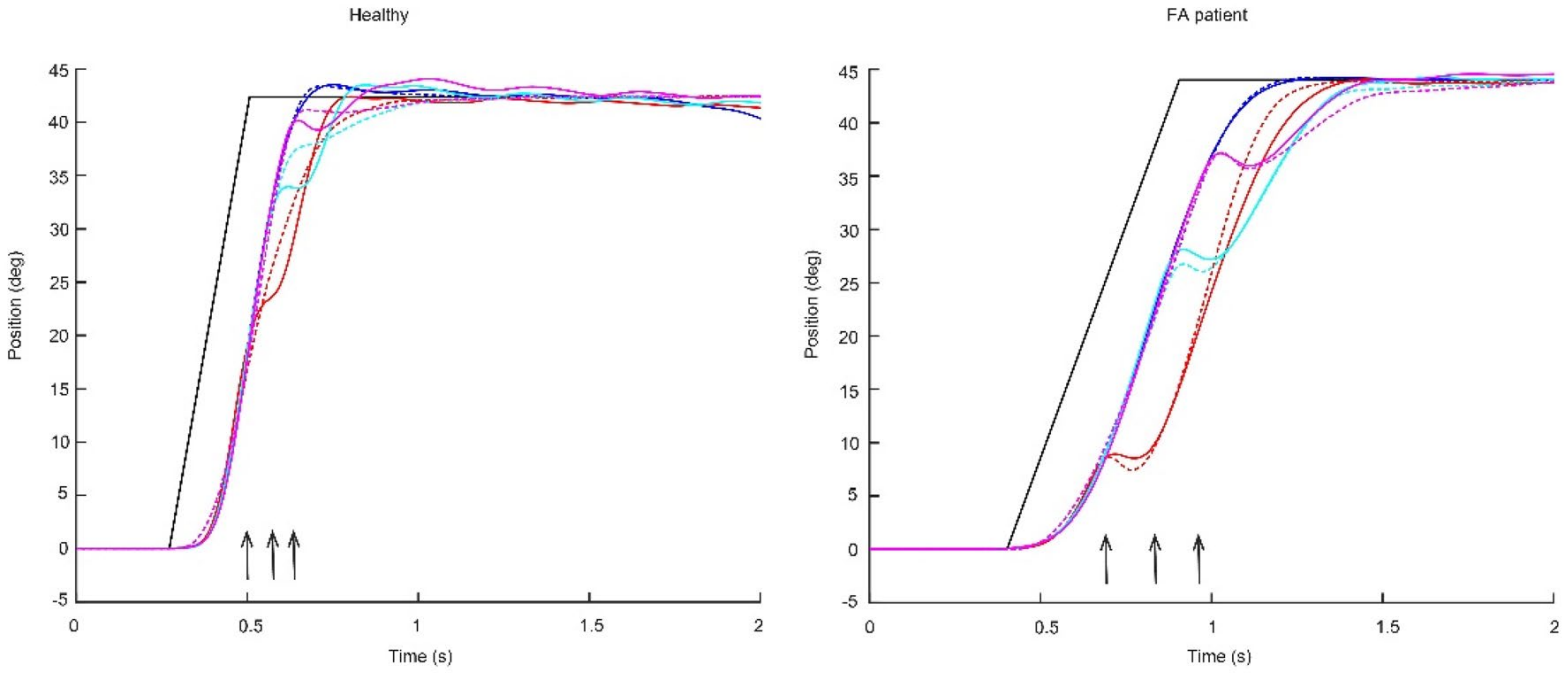
Movement kinematics (solid) and model simulation (dashed) in all conditions (no in blue/early in red/middle in cyan/late in magenta) in one sample healthy subject (left) and one FA patient (right). Vertical arrows indicate perturbation onsets (early/middle/late). The black curve is the optimized control command

**Fig. 5:**
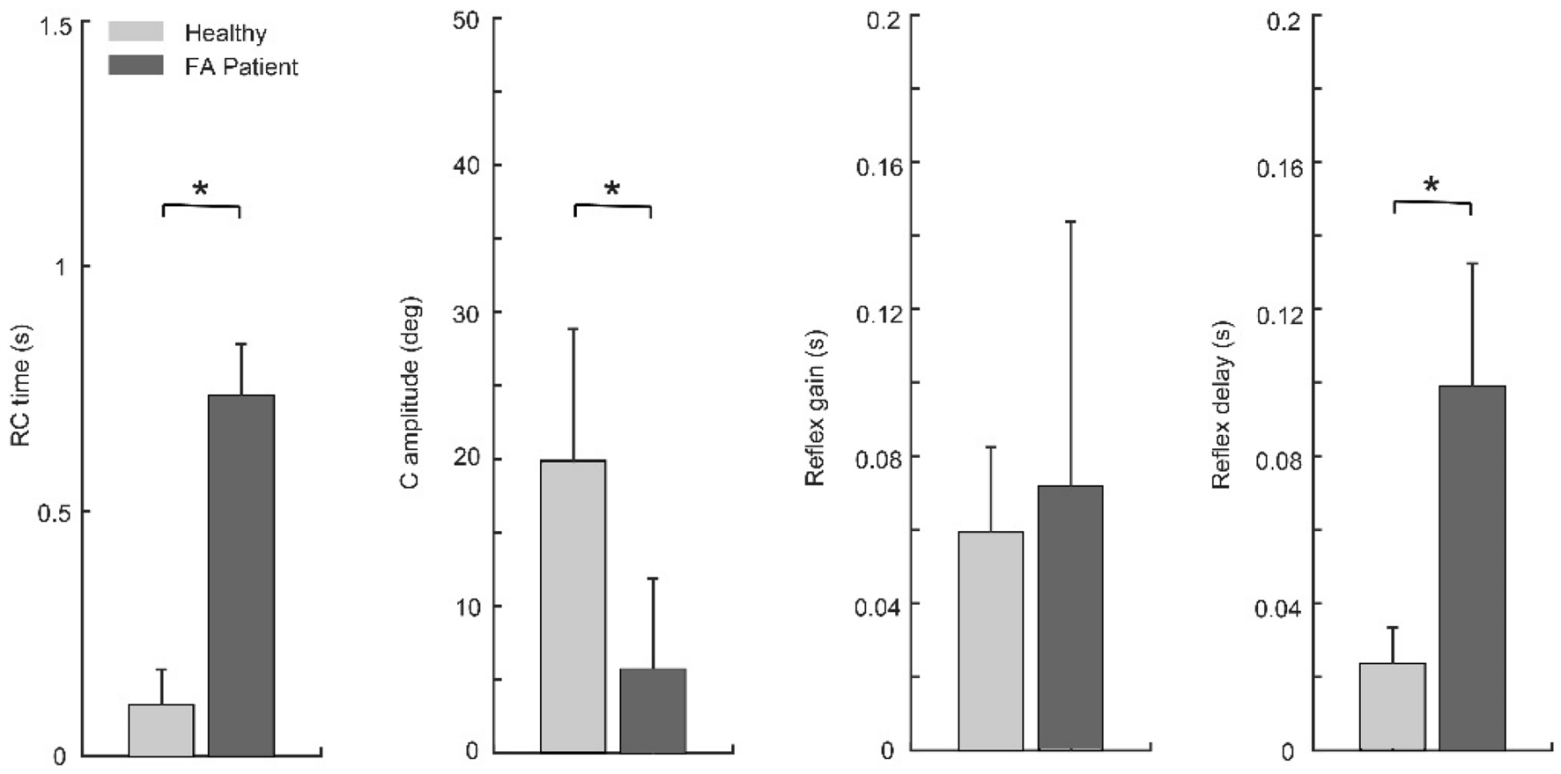
Optimized model parameters in healthy and FA groups (*n* = 6). Asterisks indicate significant differences (*t* test: *p* < .05). The increase of the RC *time* and the decrease of the C *amplitude* in FA quantify the slowdown of top-down control, whereas the increase of the reflex delay points toward a specific deficit of feedback control in these patients

Figure 4 shows the comparisons between experimental (solid curves) and simulated (dashed curves) kinematics in all conditions in one sample healthy subject (left) and one FA patient (right). The ramp-shaped referent command (black curves) effectively reproduced the joint positions in healthy and FA subjects in all perturbation conditions (for healthy: *R*^2^ = 0.997 ± 0.002, mean squared residual = 0.907 ± 0.831 deg^2; for FA: *R*^2^ = 0.983 ± 0.023, mean squared residual = 2.048 ± 1.147 deg^2). In healthy subjects, the duration of the ramp was (0.103 ± 0.074 s), which was shorter than that (0.735 ± 0.105 s) in FA patients (*p* < 0.001, Fig. 5A). The co-contraction level was higher in healthy subjects (19.877 ± 8.942 deg) than in FA patients (5.697 ± 6.173 deg, *p = *0.029, Fig. 5B). Reflex gain did not differ between the two groups (healthy: 0.059 ± 0.023, FA: 0.072 ± 0.071, *p = *0.755, Fig. 5C). Otherwise, the reflex delay of healthy subjects (0.024 ± 0.010 s) was significantly shorter than that (0.099 ± 0.033 s) of FA patients (*p = *0.002, Fig. 5D).

## Discussion

Our study shows that elbow–joint movements in FA patients preserved similar temporal profiles, such as bell-shaped velocity and bi-phasic muscle torque, as those in healthy subjects, although FA patients had significantly lower speed and smaller muscle torque than the healthy subjects. In line with this, simulation with a non-linear physiological-plausible model of movement control suggested that FA patients have a prolonged descending control signal and a lower co-contraction. Our results further demonstrated that both healthy and FA patients were able to compensate for small perturbations (max. dis 5–15°) efficiently (endpoint error < 0.5°). With larger perturbation-induced displacement, FA patients generated smaller torque responses with longer onset time to compensate for the perturbation, over a longer movement duration. Simulation results imply that this can be accounted for by a normal reflex gain amplitude and longer reflex delay in FA patients.

### Movement kinematics and kinetics compared to healthy subjects

General kinematic and kinetic profiles, such as bell-shaped velocity (Flash and Hogan 1985) and bi-phasic muscle torque were preserved in FA patients (Fig. 2). These typical movement patterns (the smooth trajectory, single peak velocity, and the acceleration peak and deceleration peak of muscle torque) rely on the efficient coordination of agonist and antagonist muscles. Thus, for the single-joint movement of the elbow, this coordination seems to be preserved. However, FA patients usually have considerable deficits when coordinating multiple joints (Cook and Giunti 2017). This may be due to a deficit in cerebellar function which is important for motor coordination (Friedemann et al. 1987). All FA patients in our study generated much slower naturalistic movements than the healthy subjects when instructed to use maximum effort. This was reflected in smaller muscle torques (comparison in Fig. 2 for a single subject and Table 1 for group comparison). The simulation showed that this may be caused by prolonged top-down control (increased duration of the rising period of the ramp-shaped control commands, i.e., *RC time*, Figs. 4 and 5). Although EMG was not measured in this study, it is reasonable to assume that FA patients have decreased muscle activity in movement generation (torque profiles). Reduced co-contraction (*C amplitude* in Fig. 5) may be another factor that leads to reduced muscle torques.

The smaller torque amplitude in unperturbed movements (comparison in Fig. 2 for a single subject and Table 1 for group comparison) implies an overall muscle weakness in FA patients. Our simulation suggests that such a weakness could be, at least partly, a result of altered central drive of the muscles (prolonged period of setting descending control signal and lower co-contraction, Fig. 5). There could be other factors that affect muscle strength, for example, those related to force generation (a and α in the third equation in Appendix 1) and those characterizing passive viscoelastic properties of the muscle (k in the fourth equation in Appendix 1). These effects may be tested in future studies.

### Compensation for small perturbations based on peripheral mechanisms

When encountering an unexpected small perturbation (as in the current study, duration < 150 ms, peak amplitude < 3Nm, for all conditions), FA patients performed generally well with resulting a small endpoint error (< 1° in response to 6–14° displacement, Fig. 3B and Fig. 3D). Notably, FA patients were also able to compensate for a larger perturbation-induced displacement (on average about 2–3 deg larger than the healthy subjects, for all conditions, Fig. 3B); however, they needed more time to do so (about 200 ms in healthy subjects and about 300 ms in FA patients, i.e., the time needed for the perturbed curve to converge to the unperturbed one, position traces in Fig. 2).

Our simulation showed that in general, reflex mechanisms may account for such compensation for small perturbations during single-joint movements, both in healthy subjects and surprisingly also in FA patients. Our results do not preclude the involvement of transcortical reactions (latency 50 ~ 100 ms, Rothwell et al. 1982). Such long-latency, supraspinal reflexes may contribute to perturbation compensation in FA patients due to the estimated time delay (*reflex delay* of FA group, Fig. 5).

A further question is why in a disease affecting the spinocerebellar tract such as in FA patients, the reflex delay but not its gain is affected. Similar results were also obtained in another study by Diener et al. (1984), who described an increased delay in the leg muscles in FA patients after perturbation. A possible factor could be that the signal-to-noise ratio of the reafference might be decreased in these patients, because of additional noise or because of the reduced number of receptors (functioning afferent nerves). Possibly, the nervous system compensates for this decreased signal-to-noise ratio by a prolonged integration time that would explain increased feedback delays.

Another question is whether the effect on movement variables and model reflex parameters was related to the age difference between the groups in our study, since we had a relatively young control group (see section Subjects in Methods). Previous studies observed that the durations of voluntary arm movements increased with age. For example, the study of Lee et al. (2007) observed that the movement duration of an arm movement was about 10% longer in a group of elderly (mean age: 68 yrs) than in a group of younger (mean age: 28 yrs) subjects. However, this difference was much smaller than the group difference of the movement duration in our study (about 184%, Table 1). Moreover, the study of Weaver et al. (2012) suggested that voluntary and perturbation-evoked reaching deteriorate similarly with age. Thus, even though the unbalanced age is not ideal in our study design, the observed group differences seem to be mainly due to the Friedreich ataxia of the patients and not to the age difference between the groups.

### Optimization approach for estimating control commands

Single-joint movements have relatively simple dynamics, such as the absence of interaction torques compared to multi-joint movements. Many studies have suggested that simple, monotonic control signals may produce single-joint movement characteristics (Pilon and Feldman 2006; St-Onge et al. 1997; Zhang et al. 2016; however, see Latash and Gottlieb 1991). In the current study, we used a ramp-shaped control command in the threshold control model. With this approach, the onset time and the duration of the ramp were considered as free parameters and estimated by an optimization technique. The same procedure was employed for essential feedback control features including reflex gain and its delay, as well as for the C command which is important for fast movements (Feldman 2015).

This optimization procedure was validated by testing with different starting points and with surrounding points. These two tests ensure that the optimization converges to a local minimum, which is critical for our optimization approach. We found that this approach is sensitive neither to starting values nor to the chosen parameter (see Appendix 2). In addition, the fitting error is very low (*R*^2^ > 0.95, Fig. 4). Thus, we are confident of the optimization accuracy and the fitting quality.

## Conclusion

Compared to healthy subjects, FA patients showed slower peak velocities, longer movement durations, and lower torque amplitudes, but, despite this scaling transformation, the shape of the temporal profiles of hand velocity and muscle torques was similar in both groups. Both FA patients and controls were able to compensate efficiently for small perturbations. The observed behavior could be accounted for in simulation by a neurophysiologically plausible model. The simulation results indicate that the patients' top-down control was altered in terms of temporal scaling, and their feedback control signal was delayed. These specific features of the motor system of FA patients, reflect the consequences of both physiological changes in proprioceptive feedback and compensatory changes in the control strategy.

## Data Availability

The data that support the findings of this study will be made available upon reasonable request to the corresponding author.

## Appendix 1

### Model description and parameter setting

A detailed schema of the model has been illustrated in a previous study (Zhang et al. 2016, its Fig. 2). Briefly, the descending commands for the flexor (λ_f_ = R + C) and the extensor (λ_e_ = R–C) are defined by the combination of R and C. Here, both descending commands are defined in units of joint angle (positive for elbow flexion). The command λ_f_ is modified by the muscle spindle feedback of the position θ and the velocity $\dot{\uptheta }$ determines the activation A_f_ of the flexor-motoneurons. λ_f_^*^ is a dynamical signal integrating different inputs to the motoneurons (i.e. descending command ${\uplambda }_{\mathrm{f}}$, velocity-dependent proprioceptive feedbacks $\upmu {\cdot \dot{\uptheta }}_{\mathrm{d}}$ and signals from reciprocal interneurons $\mathrm{r}\bullet {\mathrm{A}}_{\mathrm{e}}$):

$${\uplambda }_{\mathrm{f}}^{*}= {\uplambda }_{\mathrm{f}}-\upmu {\cdot \dot{\uptheta }}_{\mathrm{d}}+\mathrm{r}\cdot {\mathrm{A}}_{\mathrm{e}}$$

The parameter µ is the reflex gain of the velocity feedback with a unit of a second. The letter d denotes a delayed variable, in this case representing the reflex delay of $\Delta$ s:

${\uptheta }_{\mathrm{d}}=\uptheta (\mathrm{t}-\Delta )$. The parameter r is the scaling factor of reciprocal interaction, and its value is set to 0.05. ${\mathrm{A}}_{\mathrm{e}}$ denotes the extensor motoneuron activation. The flexor muscle is activated (i.e. A_f_ > 0) only if λ_f_^*^ exceeds the delayed position feedback signal θ_d_.

$${\mathrm{A}}_{\mathrm{f}}=\left\{\begin{array}{l}{\uplambda }_{\mathrm{f }}^{*}-{\uptheta }_{\mathrm{d}}, {\uptheta }_{\mathrm{d}}\le {\uplambda }_{\mathrm{f}}^{*}\\ 0 , {\uptheta }_{\mathrm{d}}>{\uplambda }_{\mathrm{f}}^{*}\end{array}\right.$$

The function of muscle torque generation in the static case is modeled by a non-linear characteristic P_f_, which is approximated by an exponential function, known as experimental invariant characteristics (Feldman and Orlovsky 1972). Parameters were set to *a* = 1.2 Nm and.

*α* = 0.045 deg^−1^.

$${\mathrm{P}}_{\mathrm{f}}=\mathrm{a}\cdot ({\mathrm{e}}^{\mathrm{\alpha }\cdot {\mathrm{A}}_{\mathrm{f}}}-1)$$

The gradual dynamic recruitment of the muscle torque M_f_ is modeled as a first-order low-pass with transfer function H(s). Muscle activations were considered proportional to the static muscle torque P_f_ (St-Onge et al. 1997). Further negative feedback due to passive mechanics of the muscle (N, Q) modifies the muscle torque to explain the total flexor torque τ_f_.

$$\mathrm{N}=\mathrm{k}\cdot {\mathrm{A}}_{\mathrm{f}}\cdot (\uptheta -{\uptheta }_{\mathrm{d}})$$

$$\mathrm{Q}=\left\{\begin{array}{l}0, {\dot{\uptheta }}_{\mathrm{d}}\ge {\mathrm{v}}_{\mathrm{m}}\\ \frac{1-\frac{1}{{\mathrm{v}}_{\mathrm{m}}}\cdot {\dot{\uptheta }}_{\mathrm{d}}}{1+\frac{1}{\mathrm{b}}\cdot {\dot{\uptheta }}_{\mathrm{d}}}, {0\le \dot{\uptheta }}_{\mathrm{d}}<{\mathrm{v}}_{\mathrm{m}}\\ \frac{1+\frac{1.3}{{\mathrm{b}}^{\mathrm{^{\prime}}}}\cdot {\dot{\uptheta }}_{\mathrm{d}}}{1+\frac{1}{{\mathrm{b}}^{\mathrm{^{\prime}}}}\cdot {\dot{\uptheta }}_{\mathrm{d}}}, {\dot{\uptheta }}_{\mathrm{d}}<0\end{array}\right.$$

The parameters were set to k = 0.008 Nm*deg^−2^; ${\mathrm{v}}_{\mathrm{m}}$=700 deg/s; b = 90 deg/s; ${\mathrm{b}}^{\mathrm{^{\prime}}}$=$-0.3\cdot \frac{{\mathrm{v}}_{\mathrm{m}}\cdot \mathrm{b}}{{\mathrm{v}}_{\mathrm{m}}+\mathrm{b}}=-24$ deg/s; *τ* = 10 ms. The difference between flexor and extensor torques equals the total muscle torque ($\tau_{m} = \tau_{f} - \tau_{e}$). All parameters in the feedback control loop were the same as those defined in Zhang et al. (2016), except that in the current study parameters µ (reflex gain) and $\Delta$ (reflex delay) are to be fitted (see the last paragraph of Methods—Simulations section).

For the simulation, we used a single-joint arm model with a total inertia of 0.1 kg m^2^, which was about the average total inertia (hand, forearm, and apparatus inertia) in our experiment. External torque for the simulation was calculated from the recorded force applied by the rope.

The control commands R and C were set to ramp signals which started to rise at movement initiation, i.e. *Start time* before recorded movement start, and reached a final constant value after a time interval of *RC time*. R started at 0 deg and terminated at the value of recorded end arm position (averaged over 100 ms after movement end) while C started at 5 deg and terminated at *C amplitude*. As mentioned above, the reflex gain was defined as *u* and the reflex delay (Δ) was defined as *Delay*.

## Appendix 2

### Testing fitting quality

We chose five variables featuring feedforward (top-down) and feedback (reflexive) control in the threshold control model. The fitted variables with their lower and upper limits and starting values (with units) are listed below:

1. *RC time* (s): 0.01 ~ 1.5/ 0.2. This variable represents the duration of top-down modulation of control commands.
2. *Start time* (s): 0.01 ~ 1/ 0.2. This variable is the start time of top-down control commands.
3. *C amplitude* (deg): – 5 ~ 50/ 10. This variable represents the level of co-contraction.
4. (s): 0.005 ~ 0.2/ 0.02. This variable is the reflex gain.
5. *u**Delay* (s): 0.01 ~ 0.2/ 0.025. This variable is the reflex delay.

We defined the cost function as the sum of square errors of position in the unperturbed and three perturbed (early, middle, late) conditions (with unit deg^2).

$$\begin{aligned} Cost & = \int _{0}^{T} [\left( {x_{{unp}}^{{fitted}} - x_{{unp}} } \right)^{2} + \left( {x_{{per\_e}}^{{fitted}} - x_{{per\_e}} } \right)^{2} \\ & + \left( {x_{{per\_m}}^{{fitted}} - x_{{per\_m}} } \right)^{2} + (x_{{per\_l}}^{{fitted}} - x_{{per\_l}} )^{2} ] \\ \end{aligned}$$

To test the quality of model fitting (e.g. if there are multiple local minima), two tests were performed to verify that this cost function was convex around the convergence point of the numerical solution. This is necessary to prove that the numerical solution provides the global optimum within a certain range of the model parameters and did not stop in a local secondary minimum. In the first test, the optimization algorithm (the Matlab function fmincon; Matlab2020b; Mathworks, Natick, MA, USA) was repeated four times using different starting values of the model parameters [*Start time*, *RC time*, *C amplitude*, *u*] shown in Table 2. All four repetitions converged at the same solution [0.30, 0.19, 32.6, 0.022]. In the second test, we plotted the cost functions along four sections keeping three of the four parameter values fixed at the optimum and varying the fourth one continuously within the following ranges: *Start time*: 0.1–0.5 s; *RC time*: 0.01–0.4 s; *C amplitude*: 12-53 deg; *u*: 0.002–0.04 s. With this procedure, no local minima were found. Thus a global minimum could be guaranteed within the tested range.

**Table 2:** The four different start points used in the optimization of the cost function

Start point	Start_time [s]	RC_time [s]	C_amp [deg]	u
1	0.3	0.1	10	0.02
2	0.25	0.2	40	0.04
3	0.01	0.01	1	0.01
4	0.5	0.5	80	0.08
